# Supplementary material for: In vitro kinase assay reveals ADP-heptose-dependent ALPK1 autophosphorylation and altered kinase activity of disease-associated ALPK1 mutants
Source: Sci Rep. 2023 Apr 18;13:6278. doi: 10.1038/s41598-023-33459-7 (PMC10113258; doi:10.1038/s41598-023-33459-7)
Supplement: Supplementary file 1 — Supplementary Figures. [file 41598_2023_33459_MOESM1_ESM.pdf]

Supplemental information

Figure S1: Raw images of the immunoblots shown in Figure 1D and quantified in 1E. For each replicate, half volume of each sample was loaded on a gel and transferred on a nitrocellulose membrane. The membrane was cut at the level of 75 kDa marker. The upper part of the membrane was blotted with an anti-myc antibody and the lower part with an anti-GST antibody. The second half of each sample was loaded on a separate gel and transferred on a membrane. The membrane was then blotted with an anti-pT9 antibody. The replicate shown in red was used for Figure 1D and the three replicates were used for quantification (red and blue rectangles).

Raw images corresponding to immunoblot data of Figure 1D and 1E of the manuscript

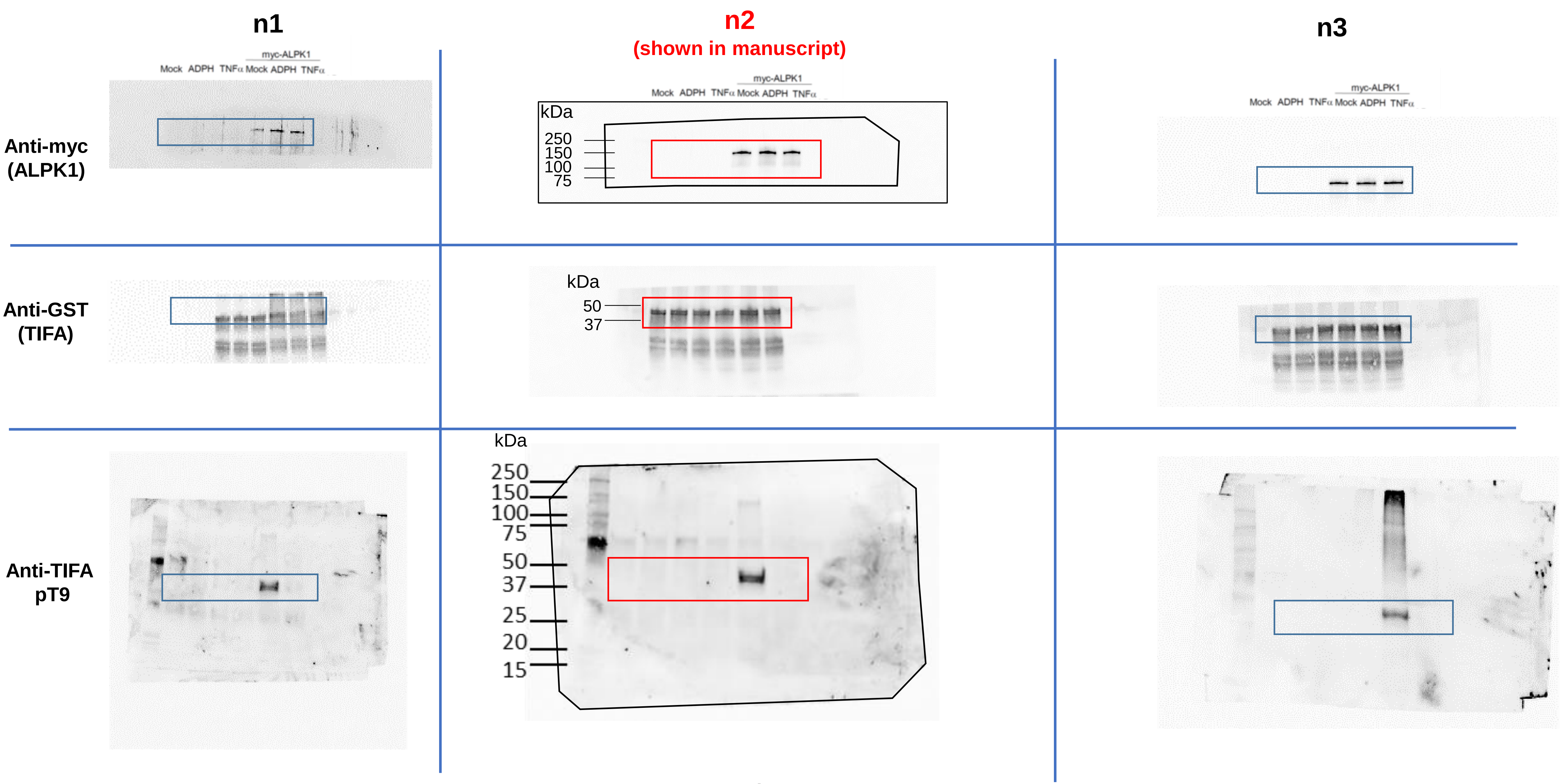

Figure S1

Figure S2: Raw images of the immunoblots shown in Figure 2A and quantified in Figure 2B. For each replicate, half volume of each sample was loaded on a gel and transferred on a nitrocellulose membrane. The membrane was cut at the level of 75 kDa marker. The upper part of the membrane was blotted with an anti-myc antibody and the lower part with an anti-GST antibody. The second half of each sample was loaded on a separate gel and transferred on a membrane. The membrane was then blotted with an anti-TE antibody.

Raw images corresponding to immunoblot data of Figure 2A and 2B of the manuscript

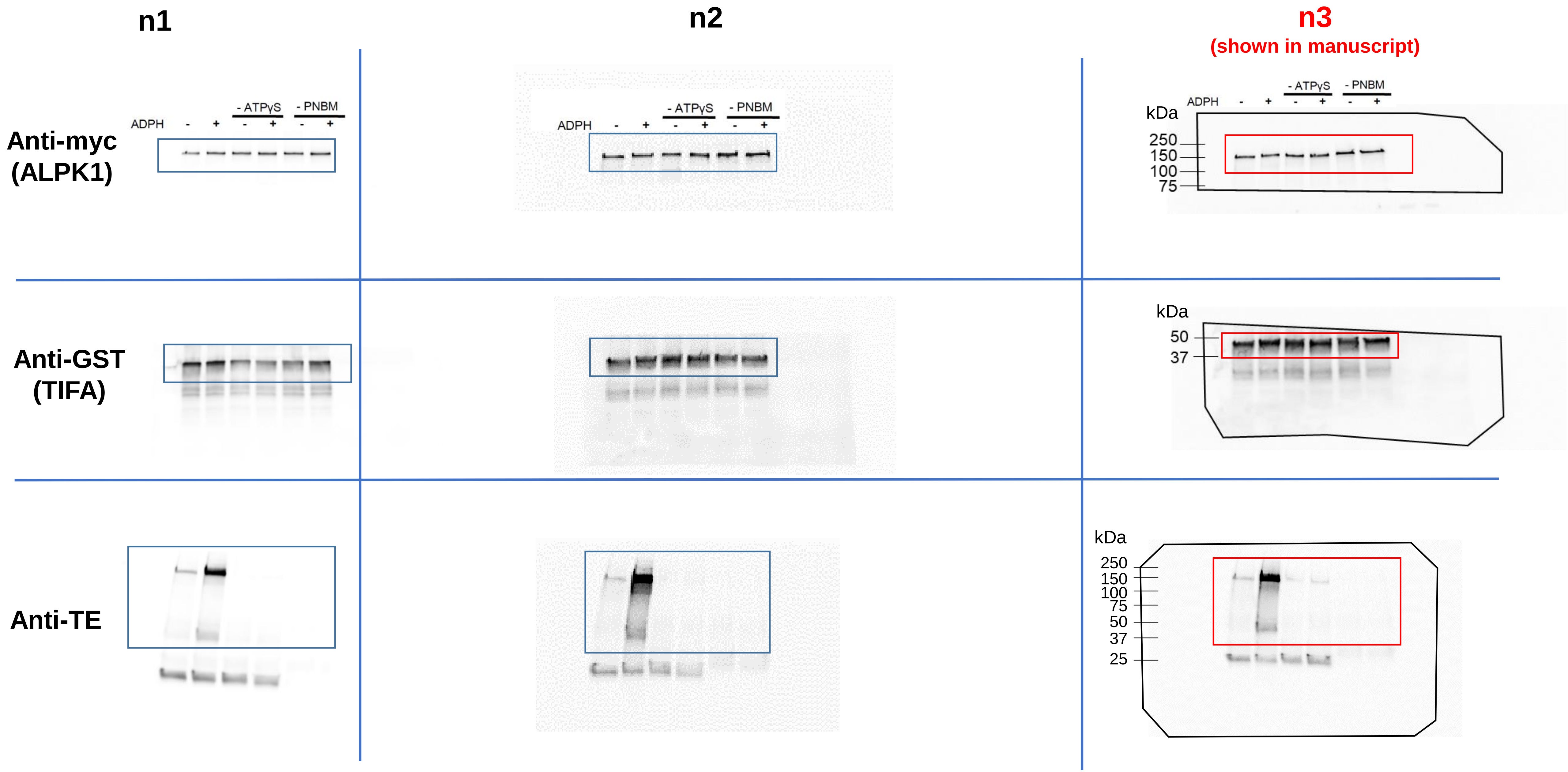

Figure S2

Figure S3: Raw images of the immunoblots shown in Figure 2C and quantified in Figure 2D. For each replicate, half volume of each sample was loaded on a gel and transferred on a nitrocellulose membrane. The membrane was cut at the level of 75 kDa marker. The upper part of the membrane was blotted with an anti-myc antibody and the lower part with an anti-GST antibody. The second half of each sample was loaded on a separate gel and transferred on a membrane. The membrane was then blotted with an anti-TE antibody. Long and short time exposures were used as indicated.

Raw images corresponding to immunoblot data of Figure 2C and 2D of the manuscript

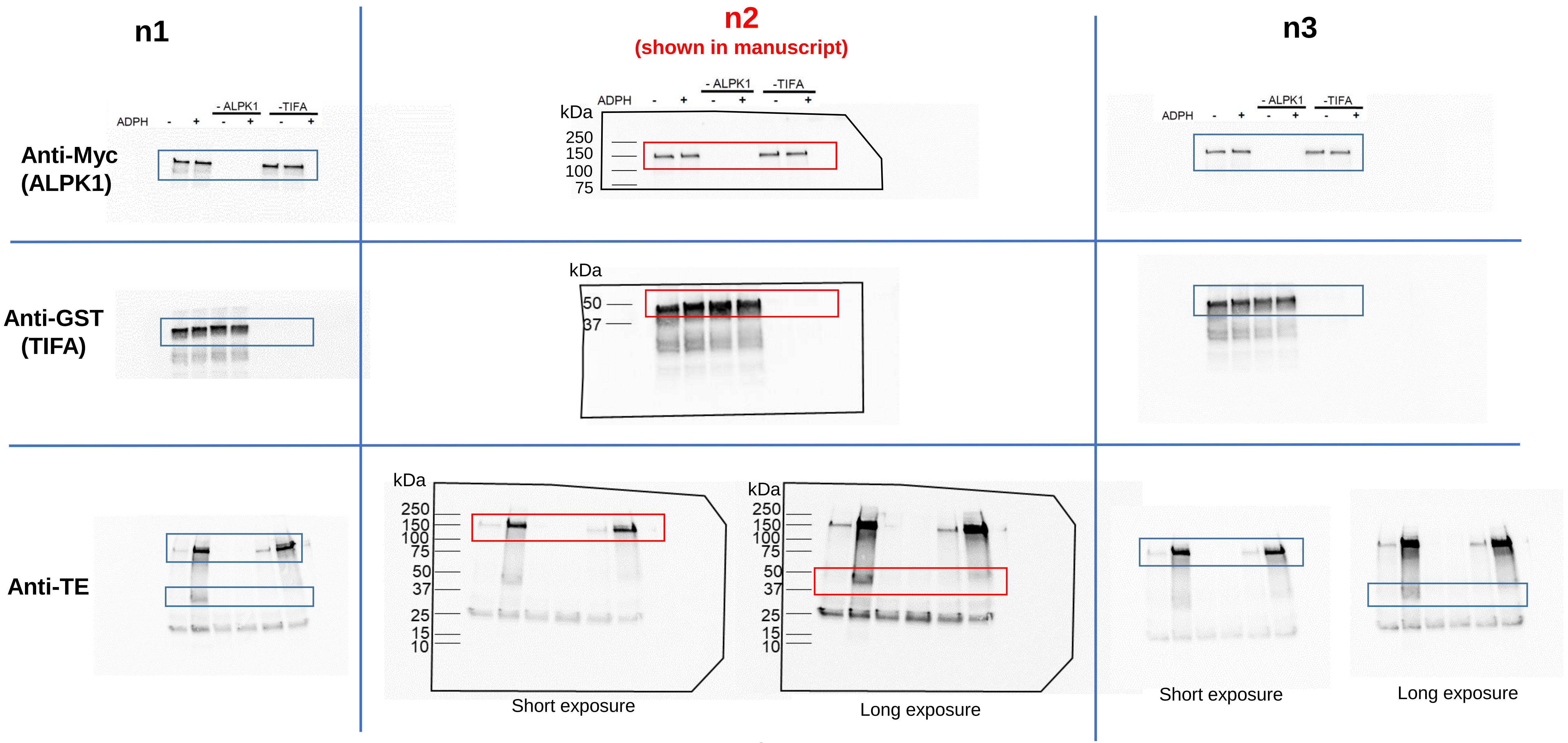

Figure S3

Figure S4: Raw images of the immunoblots shown in Figure 2E and quantified in Figure 2F. For n=1, half volume of each sample was loaded following the loading plan shown on the upper corner of Figure S5. Only the conditions shown in green were taken into account for quantification. After transfer, each membrane was cut at the level of 75 kDa marker. The upper part of each membrane was blotted with an anti-myc antibody and the lower part with an anti-GST antibody. The second half of each sample was loaded on two different gels following the same loading plan and transferred on membranes. Each membrane was then blotted with an anti-TE antibody. The first membrane was stripped and blotted again with an anti-myc. For n=2 and n=3, each sample was loaded on 3 different gels and transferred on 3 different nitrocellulose membranes. Membranes were blotted with anti-myc, anti-GST and anti-TE antibodies, respectively.

Raw images corresponding to immunoblot data of Figure 2E and 2F of the manuscript

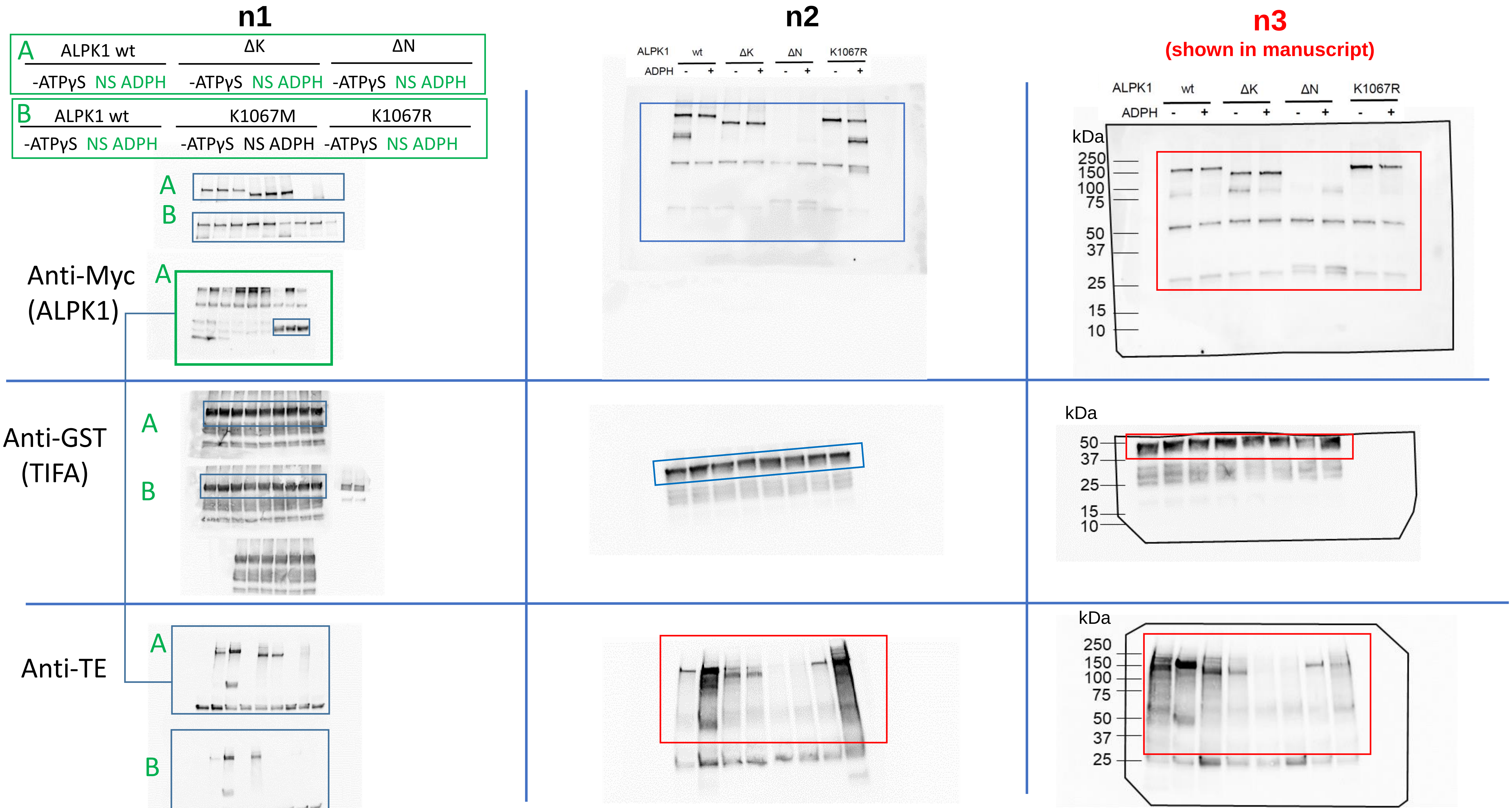

Figure S4

Figure S5: Raw images of the immunoblots shown in Figure 3A and quantified in 3B and 3C. For each replicate, half volume of each sample was loaded on a gel and transferred on a nitrocellulose membrane. The membrane was cut at the level of 75 kDa marker. The upper part of the membrane was blotted with an anti-myc antibody and the lower part with an anti-GST antibody. The second half of each sample was loaded on a separate gel and transferred on a membrane. The membrane was then blotted with an anti-TE antibody.

Raw images corresponding to immunoblot data of Figure 3A, 3B and 3C of the manuscript

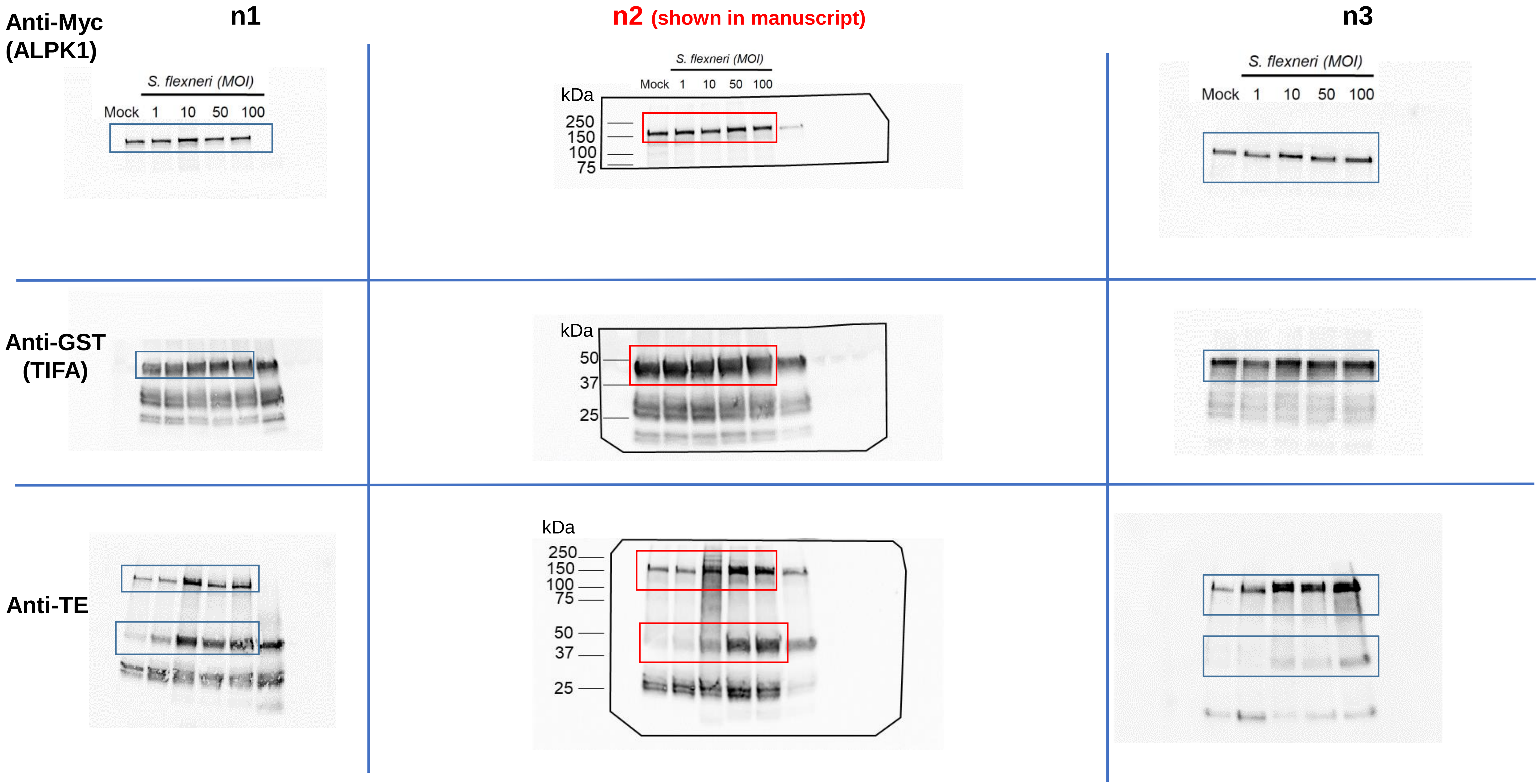

Figure S5

Figure S6: Raw images of the immunoblots shown in Figure 3D and quantified in Figure 3E. For n=1, half volume of each sample was loaded on a gel and transferred on a nitrocellulose membrane. The membrane was cut at the level of 75 kDa marker. The upper part of the membrane was blotted with an anti-myc antibody and the lower part with an anti-GST antibody. The second half of each sample was loaded on a separate gel and transferred on a membrane. The membrane was then blotted with an anti-TE antibody. For n=2 and n=3, all samples were loaded on a gel and transferred on a nitrocellulose membrane. The membrane was then blotted with an anti-TE antibody. The membrane was then stripped and cut as indicated by the scissor. The upper part of the membrane was blotted with an anti-myc antibody and the lower part with an anti-GST antibody.

Raw images corresponding to immunoblot data of Figure 3D and 3E of the manuscript

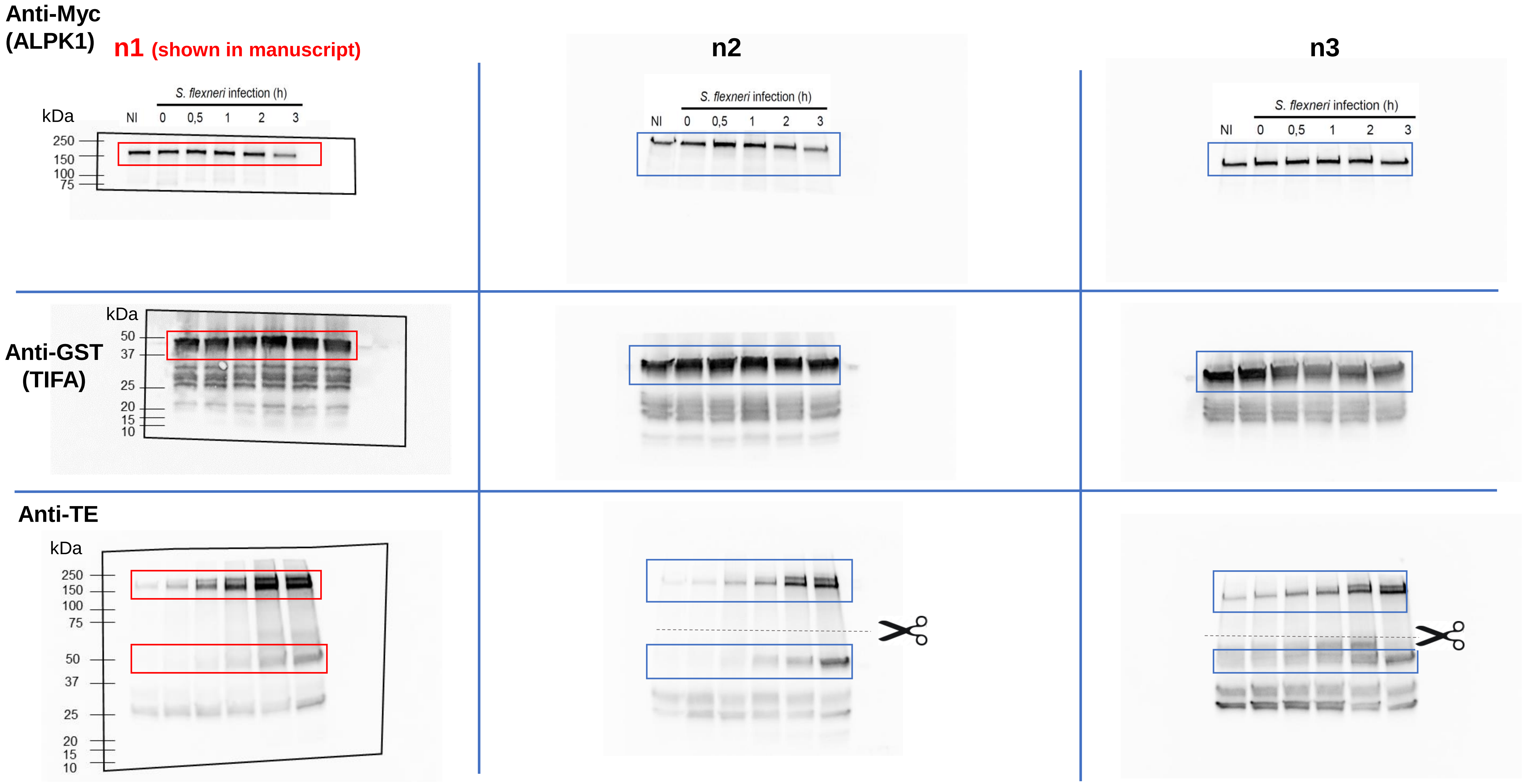

Figure S6

Figure S7: Raw images of the immunoblots shown in Figure 3F and quantified in Figure 3G and 3H. For each replicate, half volume of each sample was loaded on a gel and transferred on a nitrocellulose membrane. The membrane was cut at the level of 75 kDa marker. The upper part of the membrane was blotted with an anti-myc antibody and the lower part with an anti-GST antibody. The second half of each sample was loaded on a separate gel and transferred on a membrane. The membrane was then blotted with an anti-TE antibody.

Raw images corresponding to immunoblot data of Figure 3F, 3G and 3H of the manuscript

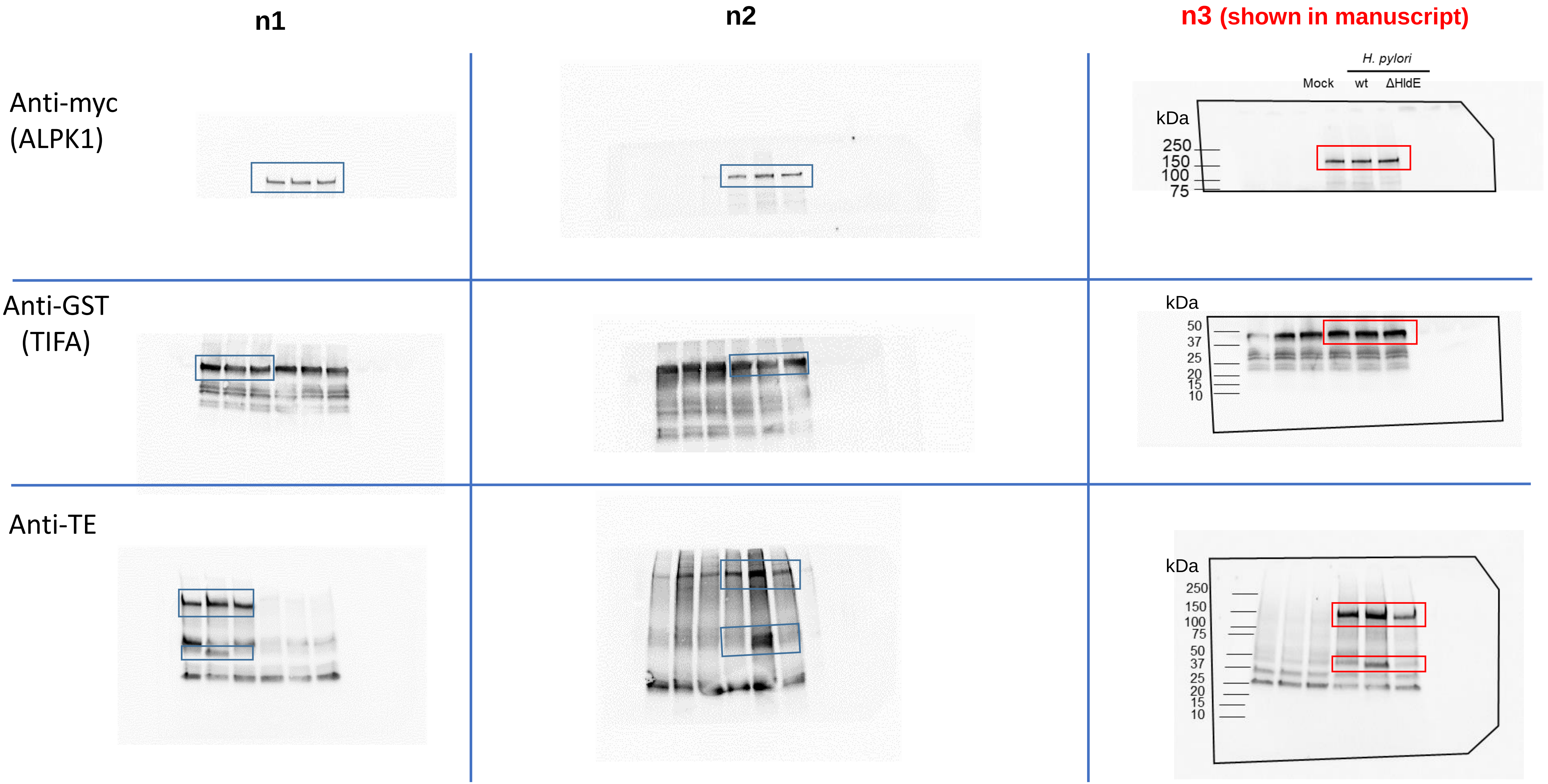

Figure S7

Figure S8: Raw images of the immunoblots shown in Figure 4A and quantified in Figure 4B. For each replicate, half volume of each sample was loaded on a gel and transferred on a nitrocellulose membrane. The membrane was cut at the level of 75 kDa marker. The upper part of the membrane was blotted with an anti-myc antibody and the lower part with an anti-GST antibody. The second half of each sample was loaded on a separate gel and transferred on a membrane. The membrane was then blotted with an anti-TE antibody. Long and short time exposures were used as indicated.

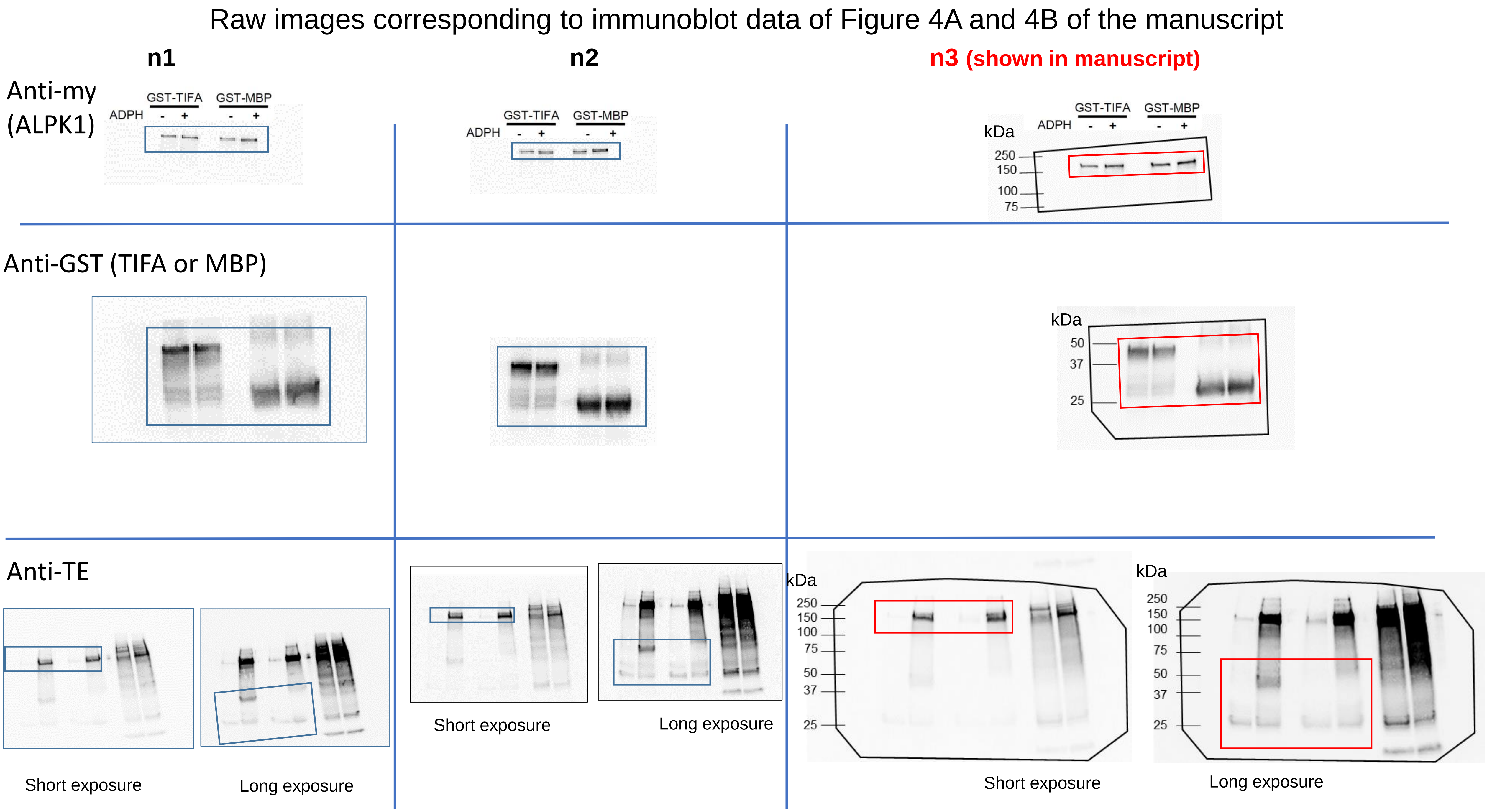
Raw images corresponding to immunoblot data of Figure 4A and 4B of the manuscript
n1
n2
n3 (shown in manuscript)
Anti-my
(ALPK1)
GST-TIFA
GST-MBP
ADPH
-
+
kDa
250
150
100
75
Anti-GST (TIFA or MBP)
50
37
25
Anti-TE
Short exposure
Long exposure

Figure S8

Figure S9: Raw images of the immunoblots shown in Figure 4C and quantified in Figure 4D and 4E. For each replicate, half volume of each sample was loaded on a gel and transferred on a nitrocellulose membrane. The membrane was cut at the level of 75 kDa marker. The upper part of the membrane was blotted with an anti-myc antibody and the lower part with an anti-GST antibody. The second half of each sample was loaded on a separate gel and transferred on a membrane. The membrane was then blotted with an anti-TE antibody.

Raw images corresponding to immunoblot data of Figure 4C, 4D and 4E of the manuscript

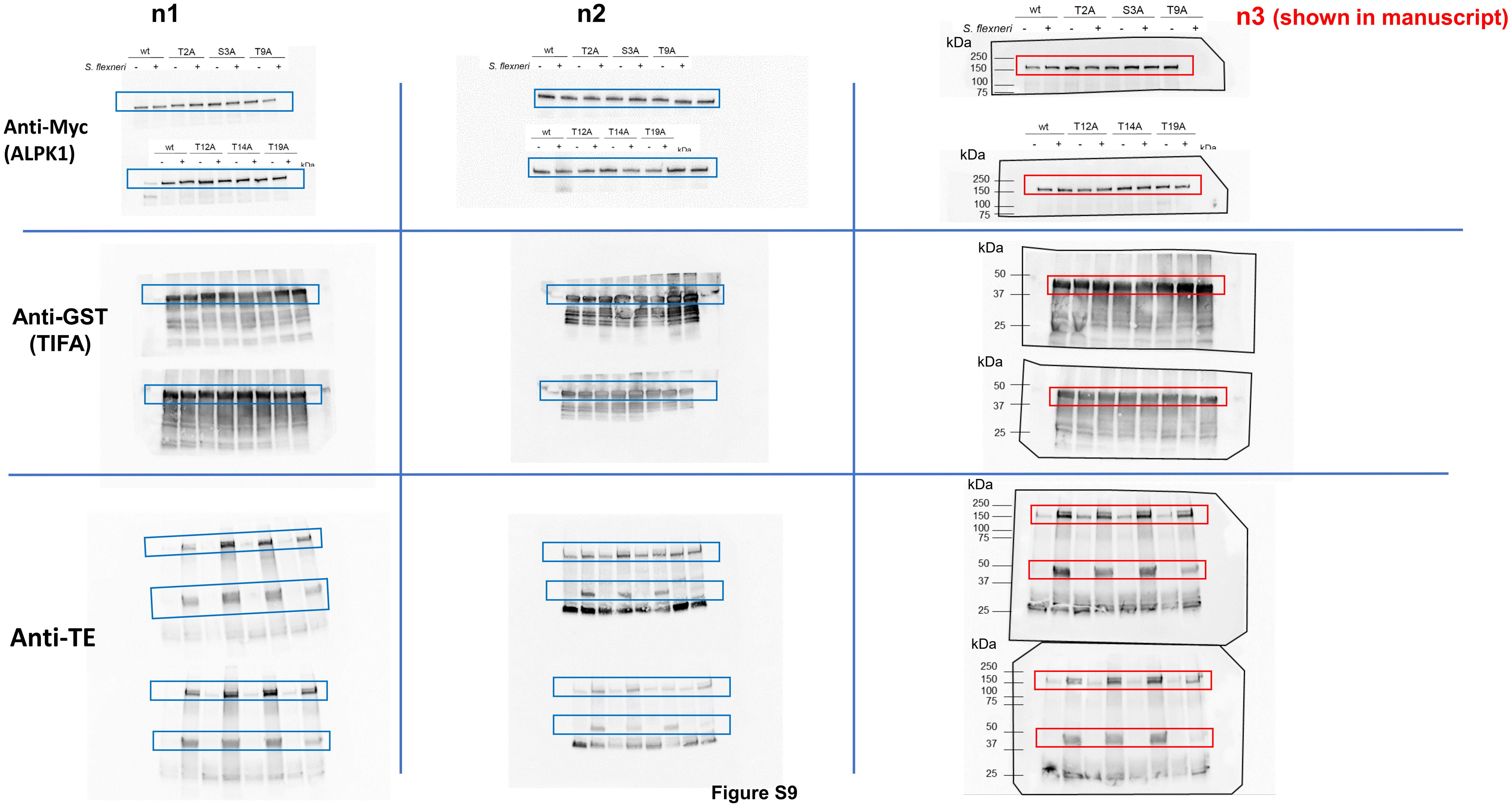

Figure S9

Figure S10: Raw images of the immunoblots shown in Figure 4F and quantified in Figure 4G. For each replicate, all samples were loaded on a gel and transferred on a nitrocellulose membrane. The membrane was then blotted with an anti-TE antibody. The membrane was then stripped and cut as indicated by the scissor. The upper part of the membrane was blotted with an anti-myc antibody and the lower part with an anti-GST antibody.

Raw images corresponding to immunoblot data of Figure 4F and 4G of the manuscript

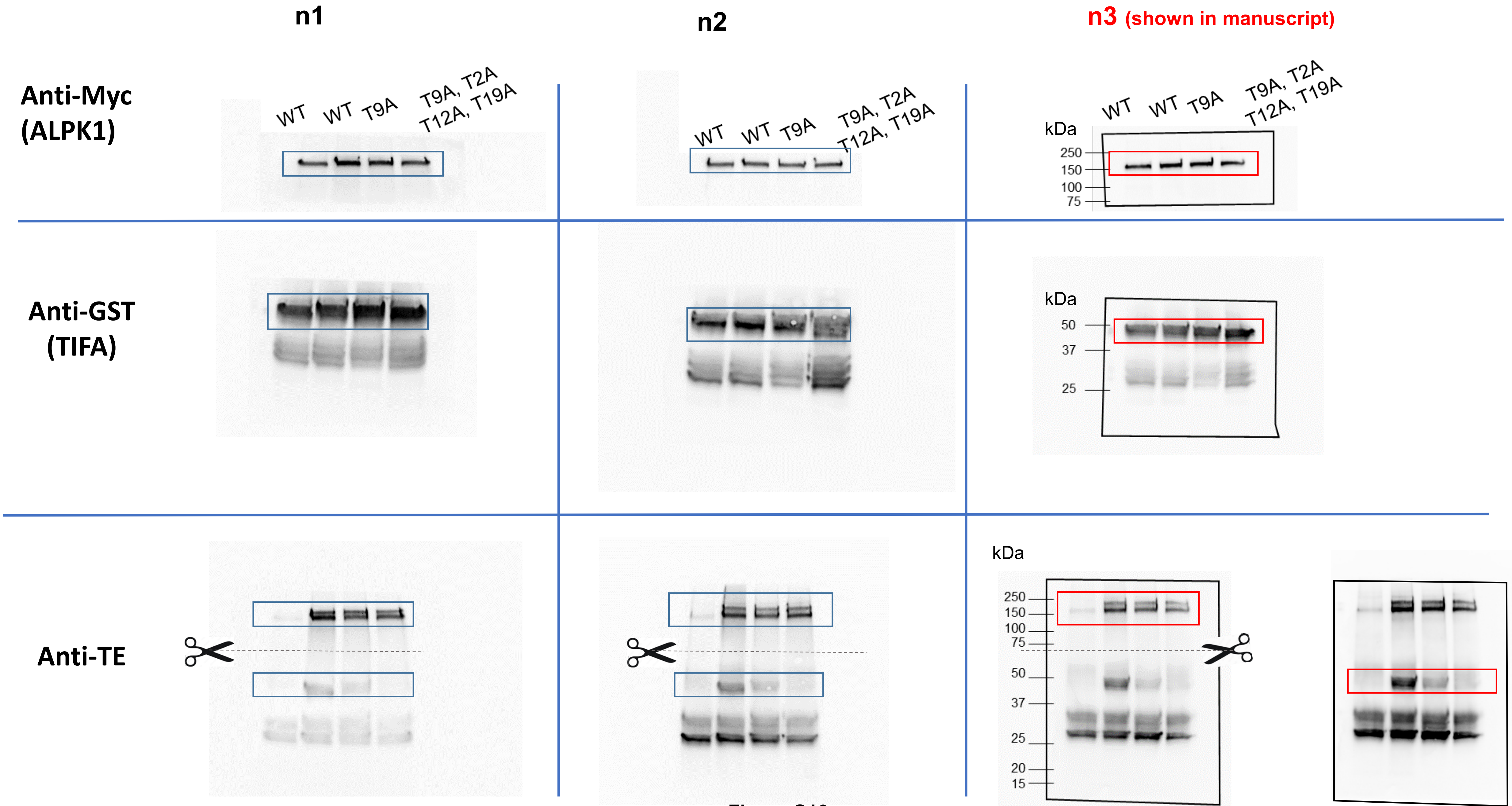

Figure S10

Figure S11: T2A, T12A and T19A TIFA mutants are able to assemble into TIFAsomes during *S. flexneri* infection. Hela cells were transfected with wt, T9A, T2A, T12A and T19A myc-TIFA constructs and infected with dsRed-expressing *S. flexneri* at MOI 10 for 1 hour. After fixation, cells were stained for myc (shown in green) and with Hoechst (shown in blue). Bacteria are shown in red. Bar is 30 μm.

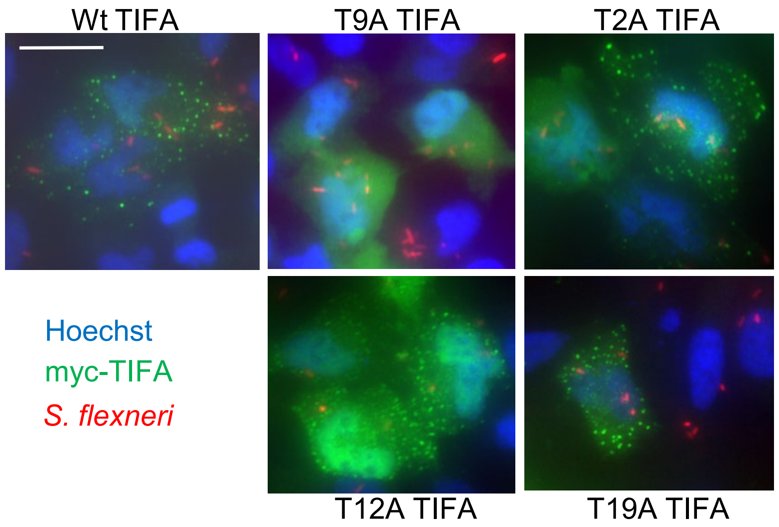
Wt TIFA
T9A TIFA
T2A TIFA
Hoechst
myc-TIFA
S. flexneri
T12A TIFA
T19A TIFA

Figure S11

Figure S12: Raw images of the immunoblots shown in Figure 5A and quantified in Figure 5B and 5C. For each replicate, samples were loaded on a gel and transferred on a nitrocellulose membrane. The membrane was then blotted with an anti-TE antibody. The membrane was then stripped and cut as indicated by the scissor. The upper part of the membrane was blotted with an anti-myc antibody and the lower part with an anti-GST antibody.

Raw images corresponding to immunoblot data of Figure 5A, 5B and 5C of the manuscript

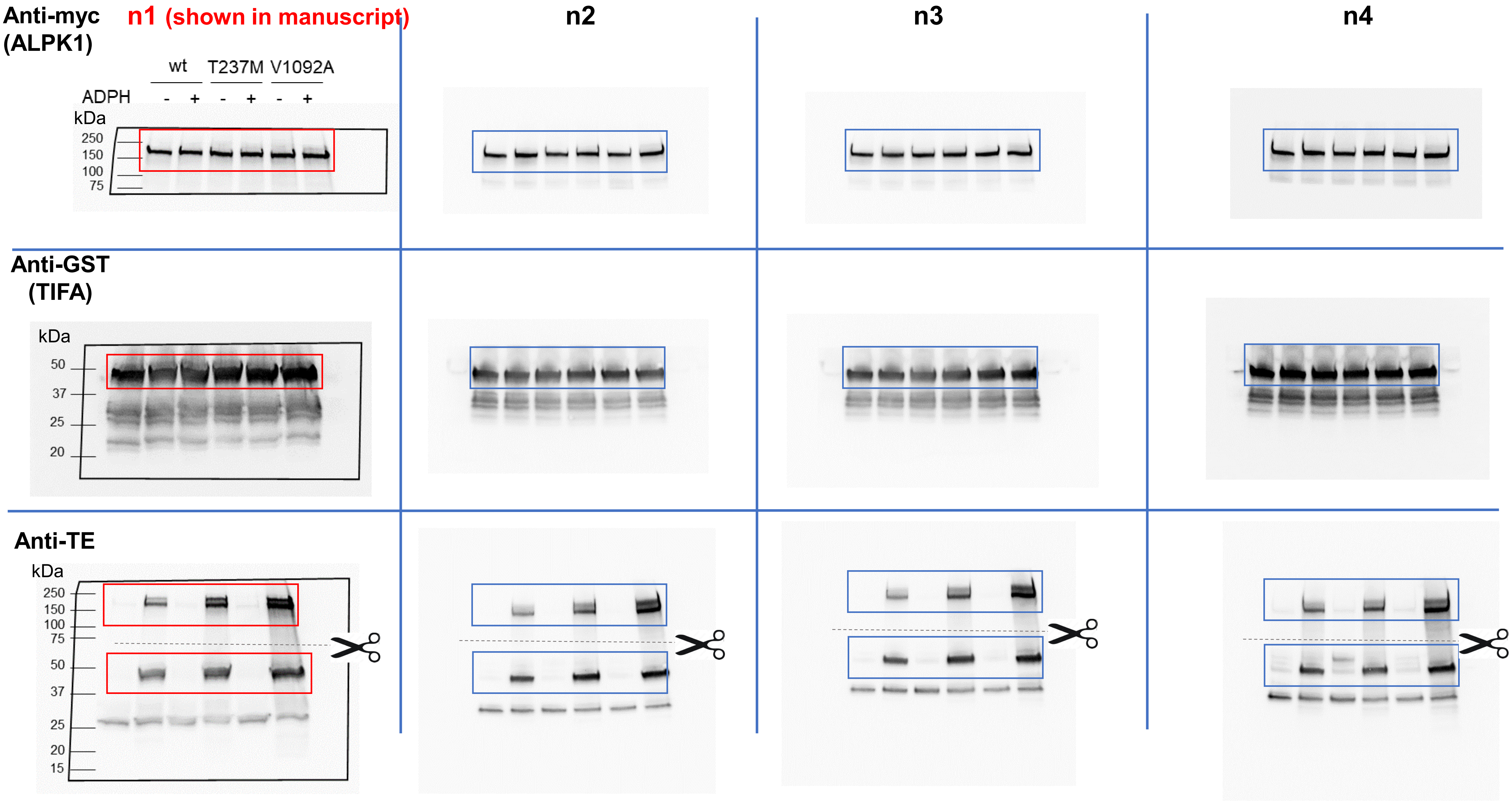

Figure S12

Figure S13: Images of the membranes corresponding to the immunoblots shown in Figure S1. For each replicate, half volume of each sample was loaded on a gel and transferred on a nitrocellulose membrane. The membrane was cut at the level of 75 kDa marker. The upper part of the membrane was blotted with an anti-myc antibody and the lower part with an anti-GST antibody. The second half of each sample was loaded on a separate gel and transferred on a membrane. The membrane was then blotted with an anti-pT9 antibody. The replicate shown in red was used for Figure 1D and the three replicates were used for quantification (red and blue rectangles).

Membrane images corresponding to the immunoblots shown in Figure S1

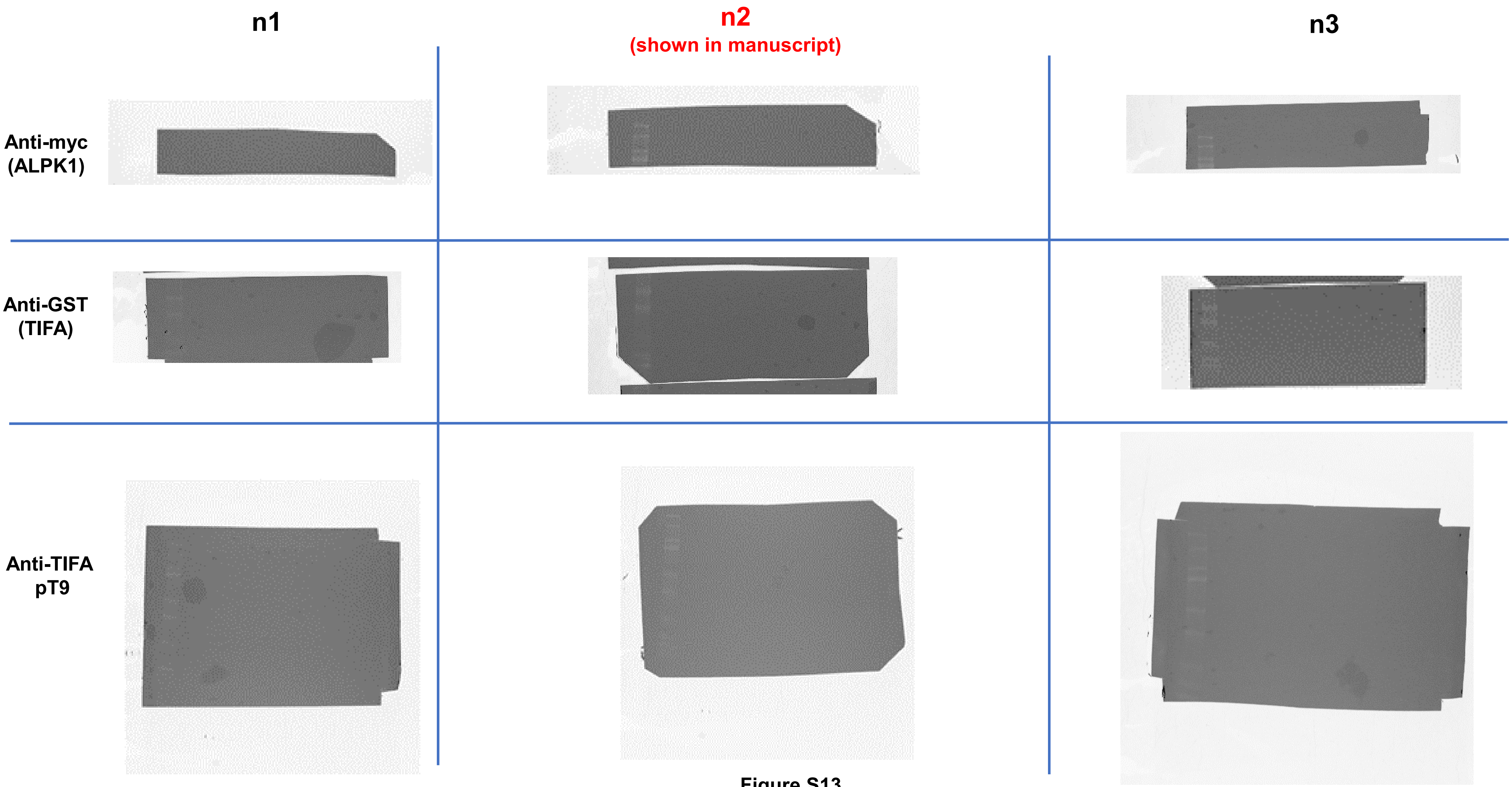

Figure S13

Figure S14: Images of the membranes corresponding to the immunoblots shown in Figure S2. For each replicate, half volume of each sample was loaded on a gel and transferred on a nitrocellulose membrane. The membrane was cut at the level of 75 kDa marker. The upper part of the membrane was blotted with an anti-myc antibody and the lower part with an anti-GST antibody. The second half of each sample was loaded on a separate gel and transferred on a membrane. The membrane was then blotted with an anti-TE antibody.

Membrane images corresponding to the immunoblots shown in Figure S2

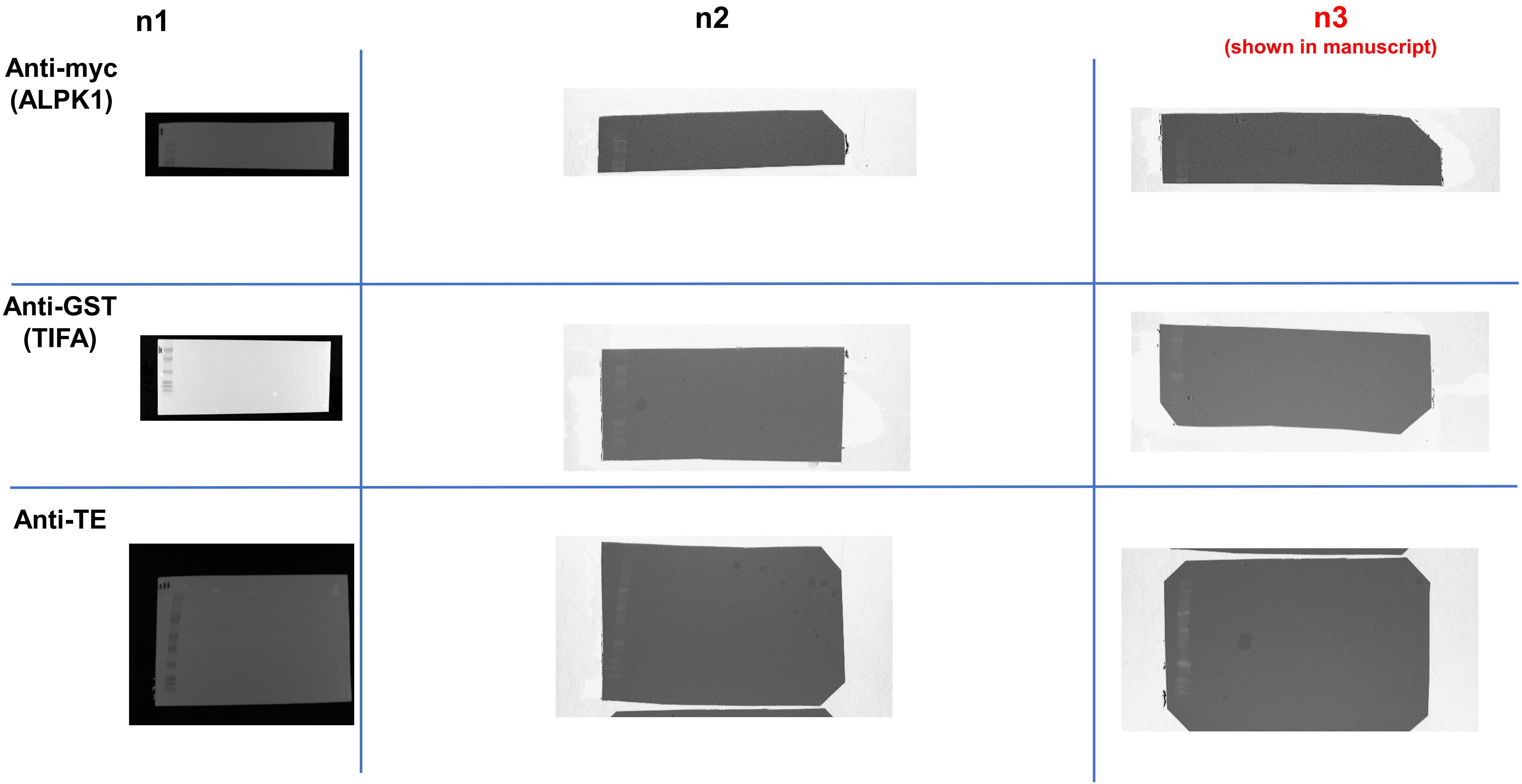

Figure S14

Figure S15: Images of the membranes corresponding to the immunoblots shown in Figure S3. For each replicate, half volume of each sample was loaded on a gel and transferred on a nitrocellulose membrane. The membrane was cut at the level of 75 kDa marker. The upper part of the membrane was blotted with an anti-myc antibody and the lower part with an anti-GST antibody. The second half of each sample was loaded on a separate gel and transferred on a membrane. The membrane was then blotted with an anti-TE antibody. Long and short time exposures were used as indicated.

Membrane images corresponding to the immunoblots shown in Figure S3

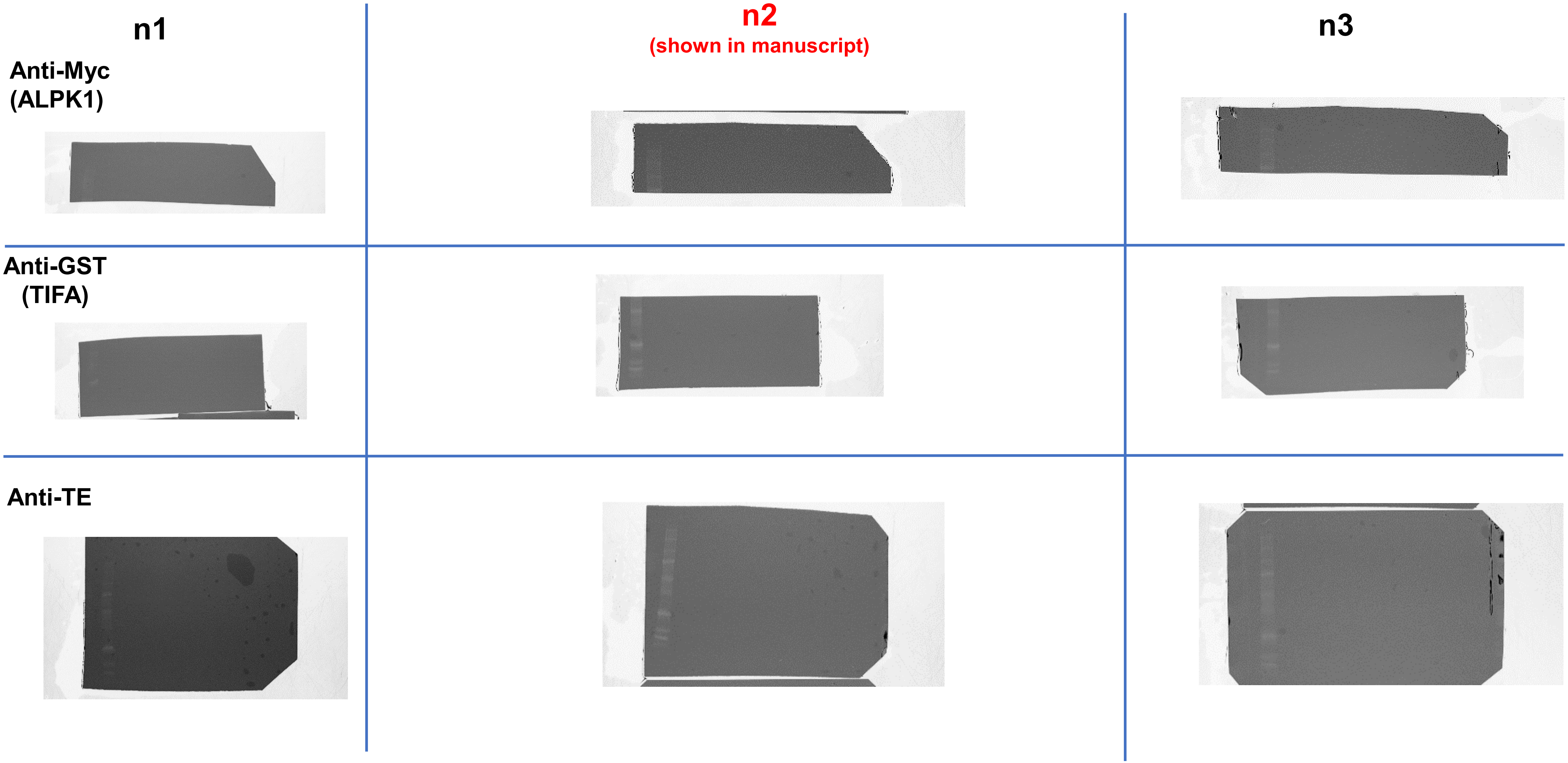

Figure S15

Figure S16: Images of the membranes corresponding to the immunoblots shown in Figure S4. For n=1, half volume of each sample was loaded following the loading plan shown on the upper corner of Figure S5. Only the conditions shown in green were taken into account for quantification. After transfer, each membrane was cut at the level of 75 kDa marker. The upper part of each membrane was blotted with an anti-myc antibody and the lower part with an anti-GST antibody. The second half of each sample was loaded on two different gels following the same loading plan and transferred on membranes. Each membrane was then blotted with an anti-TE antibody. The first membrane was stripped and blotted again with an anti-myc. For n=2 and n=3, each sample was loaded on 3 different gels and transferred on 3 different nitrocellulose membranes. Membranes were blotted with anti-myc, anti-GST and anti-TE antibodies, respectively.

Membrane images corresponding to the immunoblots shown in Figure S4

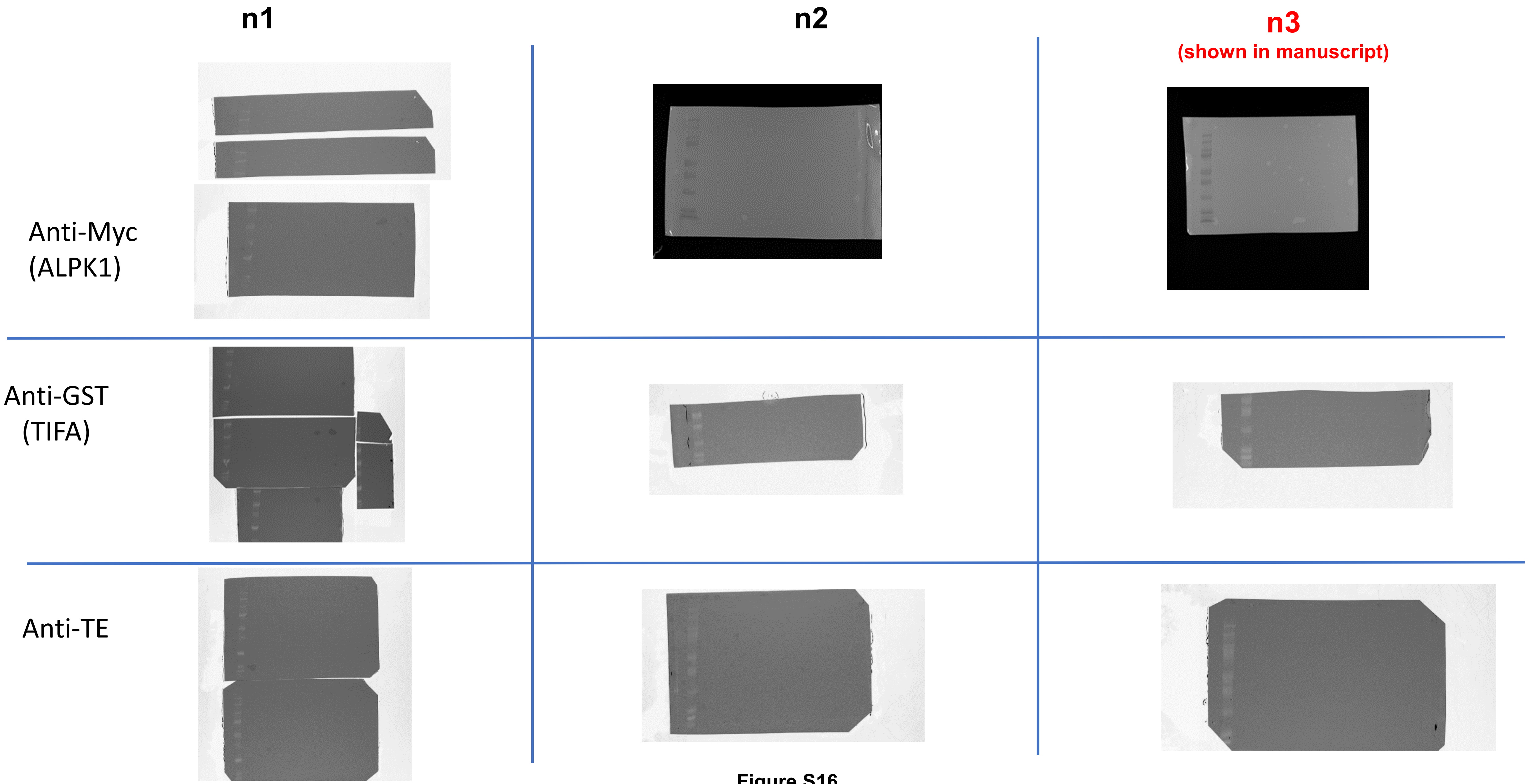

Figure S16

Figure S17: Images of the membranes corresponding to the immunoblots shown in Figure S5. For each replicate, half volume of each sample was loaded on a gel and transferred on a nitrocellulose membrane. The membrane was cut at the level of 75 kDa marker. The upper part of the membrane was blotted with an anti-myc antibody and the lower part with an anti-GST antibody. The second half of each sample was loaded on a separate gel and transferred on a membrane. The membrane was then blotted with an anti-TE antibody.

Membrane images corresponding to the immunoblots shown in Figure S5

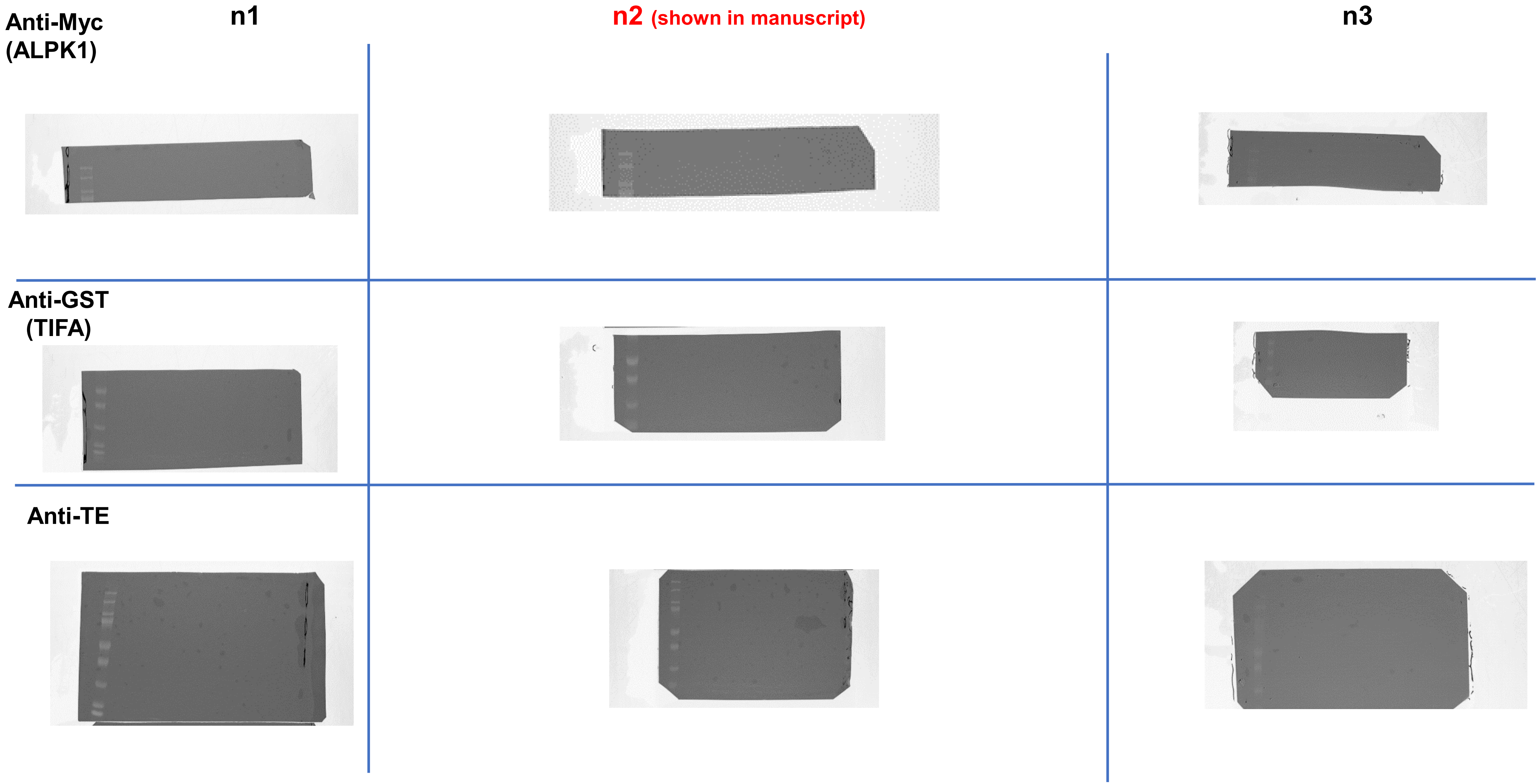

Figure S17

Figure S18: Images of the membranes corresponding to the immunoblots shown in Figure S6. For n=1, half volume of each sample was loaded on a gel and transferred on a nitrocellulose membrane. The membrane was cut at the level of 75 kDa marker. The upper part of the membrane was blotted with an anti-myc antibody and the lower part with an anti-GST antibody. The second half of each sample was loaded on a separate gel and transferred on a membrane. The membrane was then blotted with an anti-TE antibody. For n=2 and n=3, all samples were loaded on a gel and transferred on a nitrocellulose membrane. The membrane was then blotted with an anti-TE antibody. The membrane was then stripped and cut as indicated by the scissor. The upper part of the membrane was blotted with an anti-myc antibody and the lower part with an anti-GST antibody.

Membrane images corresponding to the immunoblots shown in Figure S6

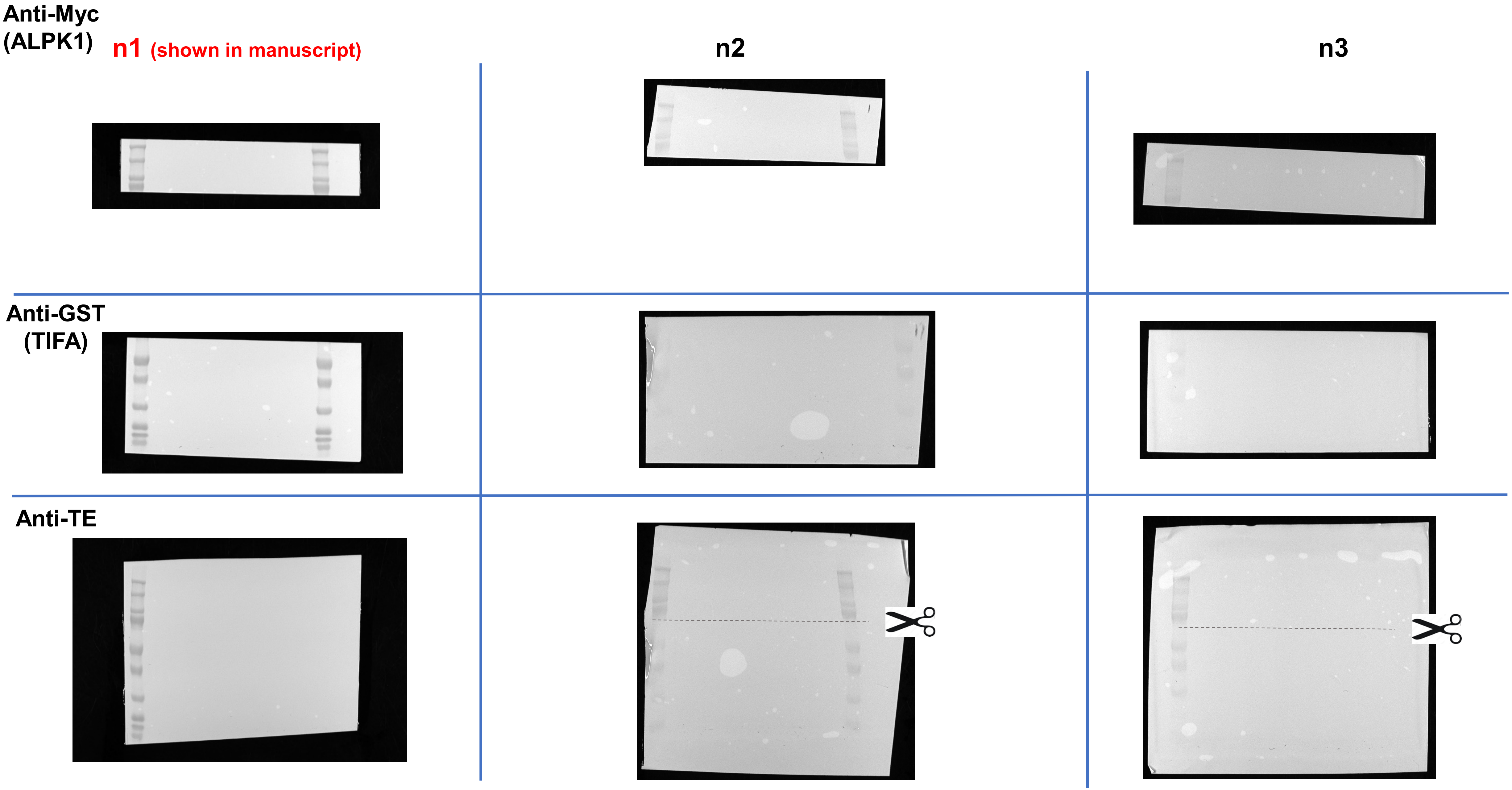

Figure S18

Figure S19: Images of the membranes corresponding to the immunoblots shown in Figure S7. For each replicate, half volume of each sample was loaded on a gel and transferred on a nitrocellulose membrane. The membrane was cut at the level of 75 kDa marker. The upper part of the membrane was blotted with an anti-myc antibody and the lower part with an anti-GST antibody. The second half of each sample was loaded on a separate gel and transferred on a membrane. The membrane was then blotted with an anti-TE antibody.

Membrane images corresponding to the immunoblots shown in Figure S7

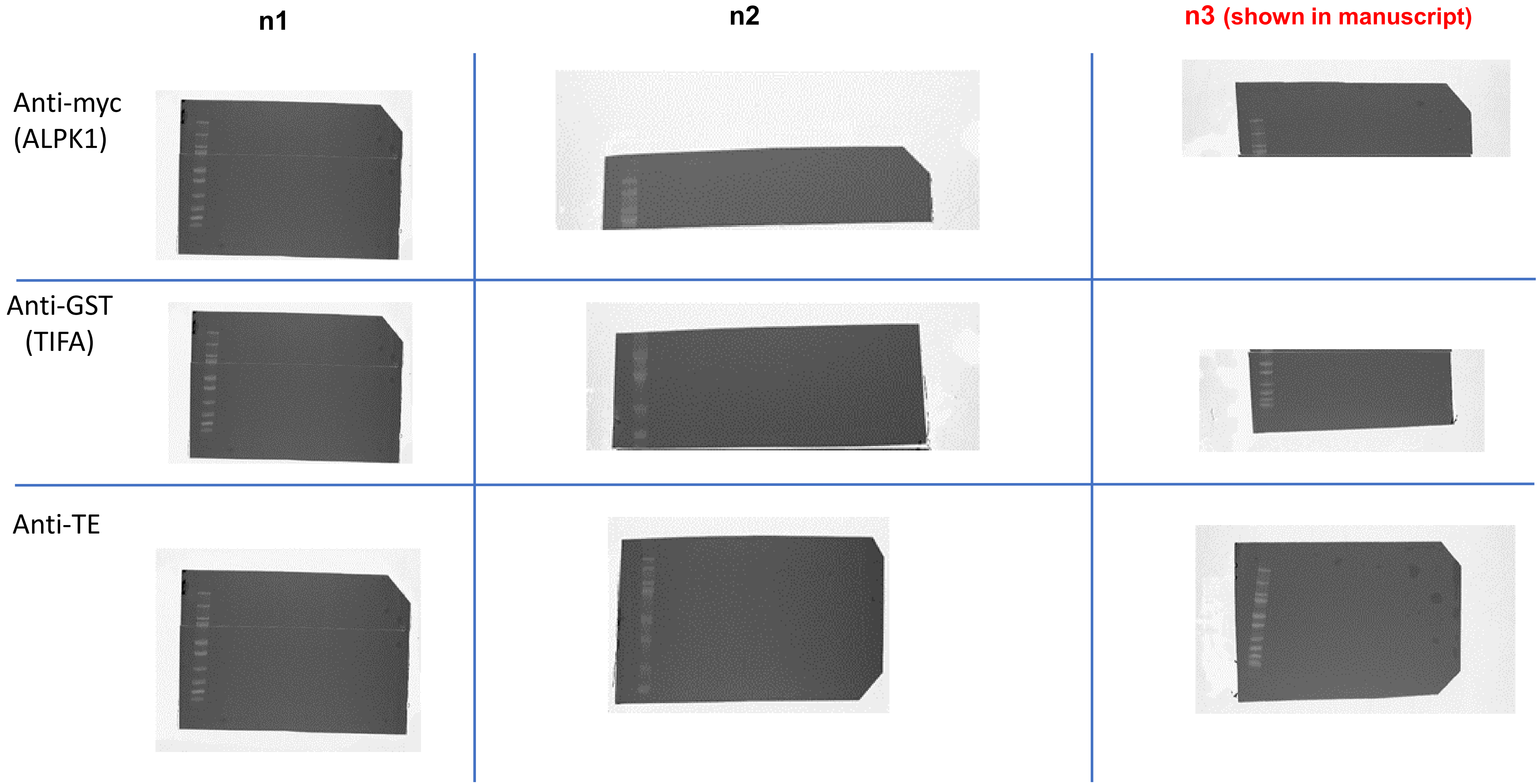

Figure S19

Figure S20: Images of the membranes corresponding to the immunoblots shown in Figure S8. For each replicate, half volume of each sample was loaded on a gel and transferred on a nitrocellulose membrane. The membrane was cut at the level of 75 kDa marker. The upper part of the membrane was blotted with an anti-myc antibody and the lower part with an anti-GST antibody. The second half of each sample was loaded on a separate gel and transferred on a membrane. The membrane was then blotted with an anti-TE antibody. Long and short time exposures were used as indicated.

Membrane images corresponding to the immunoblots shown in Figure S8

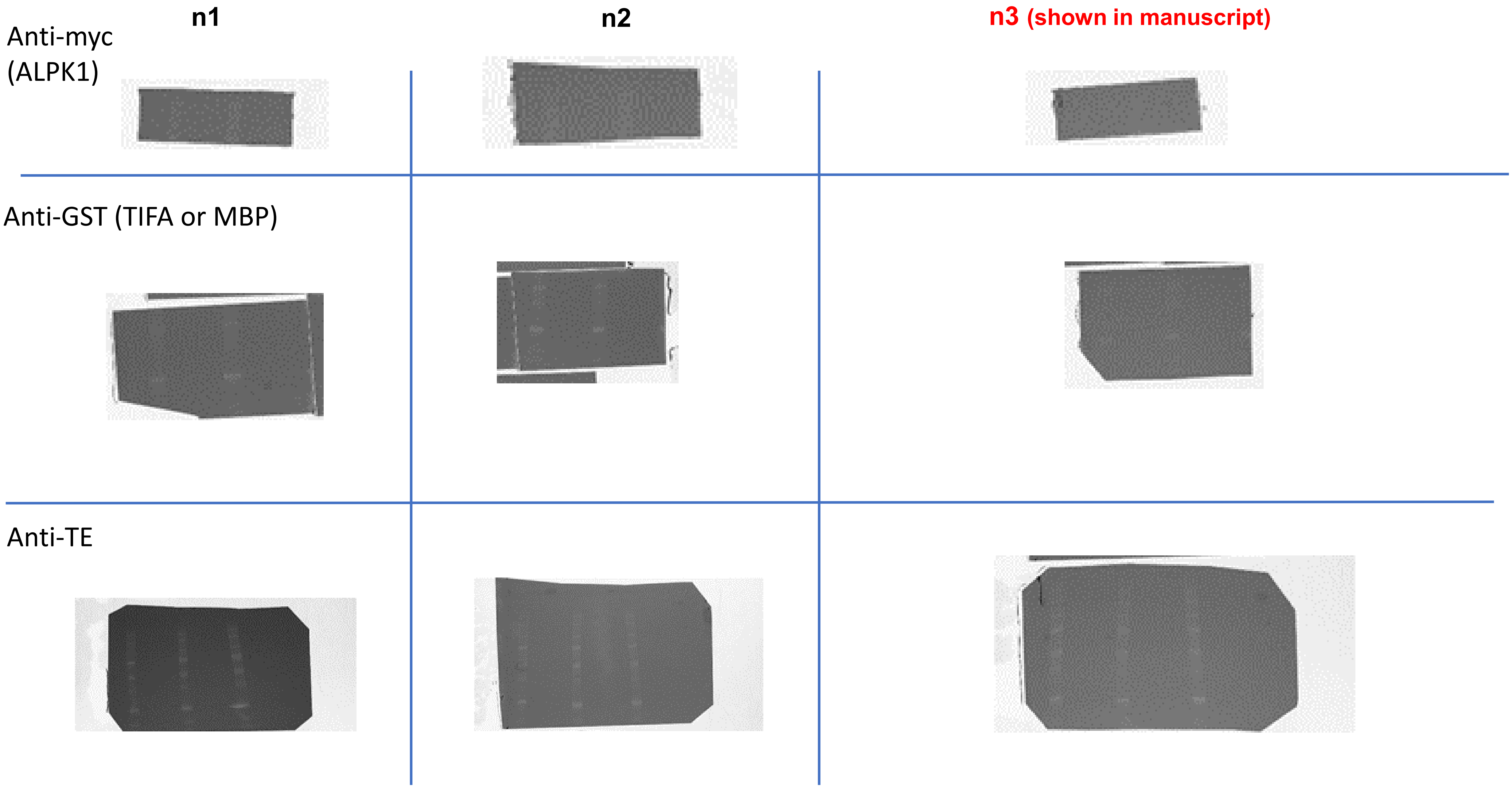

Figure S20

Figure S21: Images of the membranes corresponding to the immunoblots shown in Figure S9. For each replicate, half volume of each sample was loaded on a gel and transferred on a nitrocellulose membrane. The membrane was cut at the level of 75 kDa marker. The upper part of the membrane was blotted with an anti-myc antibody and the lower part with an anti-GST antibody. The second half of each sample was loaded on a separate gel and transferred on a membrane. The membrane was then blotted with an anti-TE antibody.

Membrane images corresponding to the immunoblots shown in Figure S9

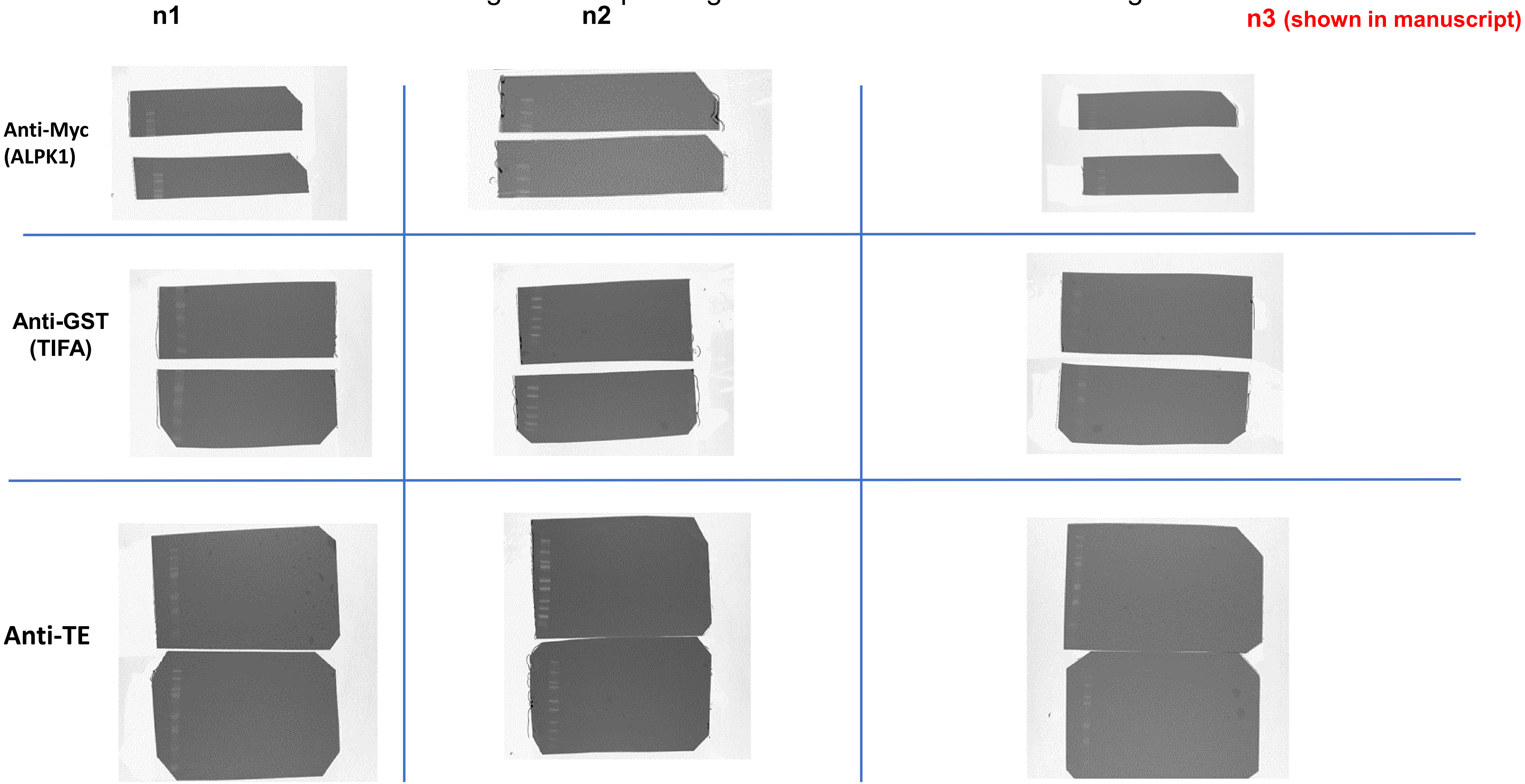

Figure S21

Figure S22: Images of the membranes corresponding to the immunoblots shown in Figure S10. For each replicate, all samples were loaded on a gel and transferred on a nitrocellulose membrane. The membrane was then blotted with an anti-TE antibody. The membrane was then stripped and cut as indicated by the scissor. The upper part of the membrane was blotted with an anti-myc antibody and the lower part with an anti-GST antibody.

Membrane images corresponding to the immunoblots shown in Figure S10

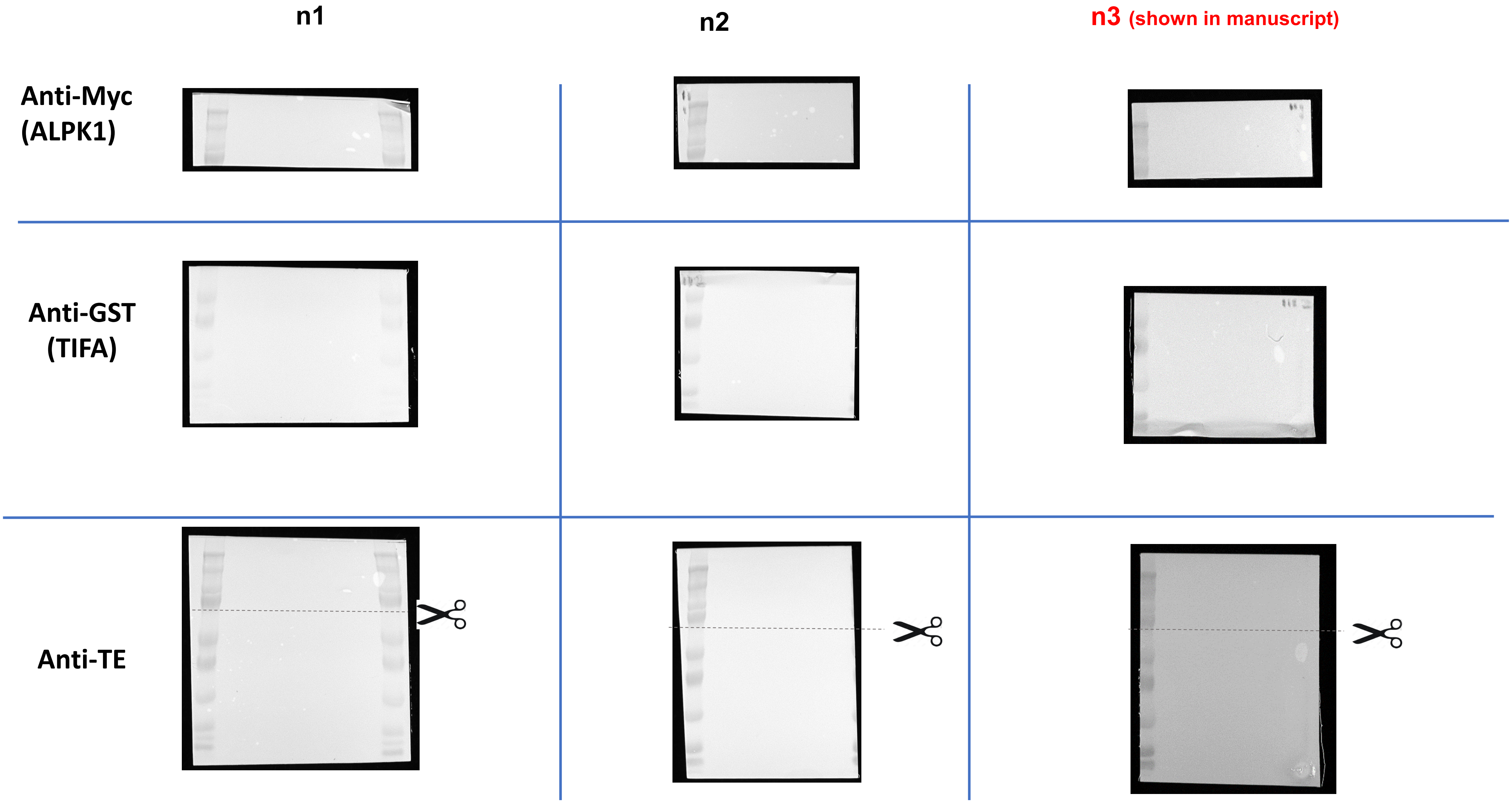

Figure S22

Figure S23: Images of the membranes corresponding to the immunoblots shown in Figure S12. For each replicate, samples were loaded on a gel and transferred on a nitrocellulose membrane. The membrane was then blotted with an anti-TE antibody. The membrane was then stripped and cut as indicated by the scissor. The upper part of the membrane was blotted with an anti-myc antibody and the lower part with an anti-GST antibody.

Membrane images corresponding to the immunoblots shown in Figure S12

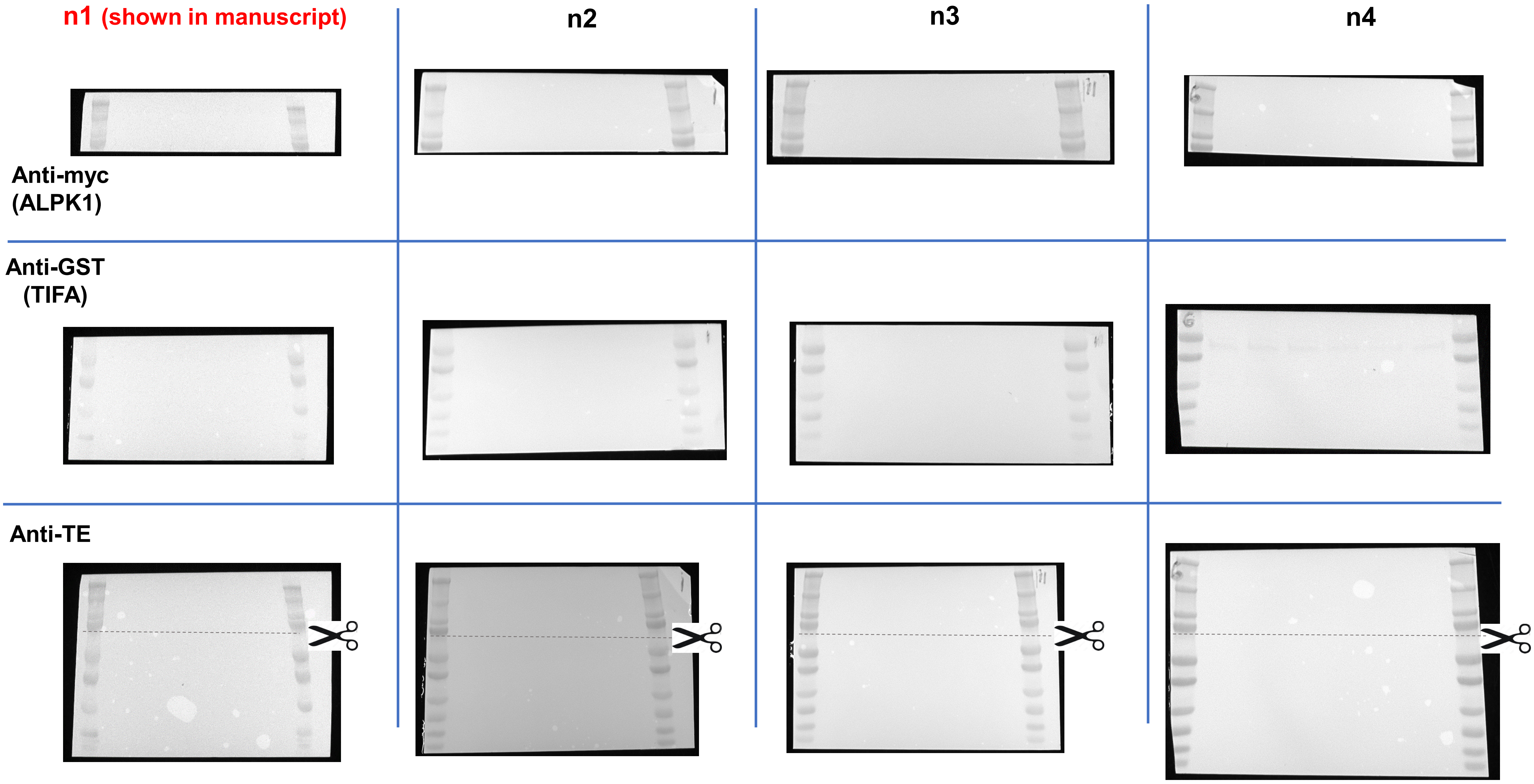

Figure S23
